# Mechanistic and Structural Insights on the IL-15 System through Molecular Dynamics Simulations

**DOI:** 10.3390/molecules24183261

**Published:** 2019-09-06

**Authors:** Rui P. Sousa, Adèle D. Laurent, Agnès Quéméner, Erwan Mortier, Jean-Yves Le Questel

**Affiliations:** 1 Université de Nantes, CEISAM UMR 6230, UFR des Sciences et des Techniques, 2 rue de la Houssinière, BP 92208, F-44000 Nantes, France; rui.sousa@univ-nantes.fr (R.P.S.); Adele.Laurent@univ-nantes.fr (A.D.L.); 2 CRCINA, CNRS, Inserm, Université d’Angers, Université de Nantes, F-44200 Nantes, France; agnes.quemener@univ-nantes.fr (A.Q.); erwan.mortier@inserm.fr (E.M.); 3 Immunotherapy, Graft, Oncology (IGO) LabEx, F-44000 Nantes, France; 4 Nantes Université, CHU Nantes, Inserm, CNRS, SFR Santé, FED 4203, Inserm UMS 016, CNRS UMS 3556, IMPACT Platform, F-44000 Nantes, France

**Keywords:** interleukin 15, IL-15 interfaces, molecular dynamics simulations, protein protein interactions

## Abstract

Interleukin 15 (IL-15), a four-helix bundle cytokine, is involved in a plethora of different cellular functions and, particularly, plays a key role in the development and activation of immune responses. IL-15 forms receptor complexes by binding with IL-2Rβ- and common γ (γc)-signaling subunits, which are shared with other members of the cytokines family (IL-2 for IL-2Rβ- and all other γc- cytokines for γc). The specificity of IL-15 is brought by the non-signaling α-subunit, IL-15Rα. Here we present the results of molecular dynamics simulations carried out on four relevant forms of IL-15: its monomer, IL-15 interacting individually with IL-15Rα (IL-15/IL-15Rα), with IL-2Rβ/γc subunits (IL-15/IL-2Rβ/γc) or with its three receptors simultaneously (IL-15/IL-15Rα/IL-2Rβ/γc). Through the analyses of the various trajectories, new insights on the structural features of the interfaces are highlighted, according to the considered form. The comparison of the results with the experimental data, available from X-ray crystallography, allows, in particular, the rationalization of the importance of IL-15 key residues (e.g., Asp8, Lys10, Glu64). Furthermore, the pivotal role of water molecules in the stabilization of the various protein-protein interfaces and their H-bonds networks are underlined for each of the considered complexes.

## 1. Introduction

Interleukin 15 (IL-15) is a cytokine targeting a plethora of cells and participating in a different number of cellular functions. Indeed, it is essential to the function and homeostasis of T lymphocytes and natural killer (NK) cells [1]. As such, it participates in the development and activation of immune responses. More precisely, its functions include the stimulation of memory T cells and NK cell proliferation, survival and activation, as well as the inhibition of apoptosis of immune cells [2].

IL-15 is a four-helix bundle cytokine, belonging to the cytokine family sharing the common gamma (γc) chain receptor, which also includes IL-2, IL-4, IL-7, IL-9, and IL-21. Among this family, IL-2 is structurally similar to IL-15, as both are the only cytokines to possess three receptor subunits, contrary to the remaining members of the four-helix bundle family, which only possess two receptor chains. Despite these two interleukins have shared functions, they are not fully functionally redundant, and can even display competing effects [3,4,5]. Based on the noticeable role of IL-15 and IL-2 in various immune system responses, intensive research, aimed at the development of novel therapies that could exploit those functional differences [6,7], has been conducted.

**Figure 1:**
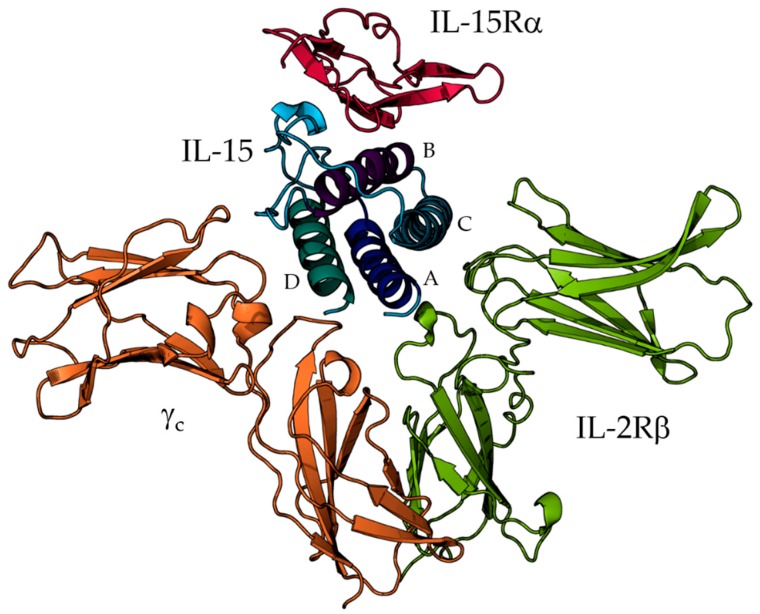
Structure of IL-15 in the full (IL-15/IL-15Rα/IL-2Rβ/γ_c_) form. IL-15 is colored in blue and the A-D helices are indicated, IL-15Rα in magenta, IL-2β in green and γc in orange.

From a structural point of view, the interleukin four-α-helix core (helices A–D, Figure 1) is hydrophobic, whereas the exposed surface is mainly constituted of polar amino acids making contacts with the different receptor chains. Both IL-2 and IL-15 bind heterodimers formed by the IL-2Rβ and the γ_c_ chains, inducing the activation of the JAK/Stat pathways [8]. Each of the cytokines has its own specific α-receptor subunit, namely, IL-2Rα and IL-15Rα, respectively. The IL-15Rα chain is a single transmembrane protein, whose distal domain, also called sushi domain, is required for IL-15 binding [9]. IL-15Rα can be expressed depending on the activation status of the cells. Thus, IL-15 can bind to the IL-2Rβ/γ_c_ dimeric receptor as well as to a high affinity IL-15Rα/IL-2Rβ/γ_c_ trimeric receptor. IL-15Rα can also be expressed by an IL-15 producing cell (macrophages, dendritic cells, epithelial cells). In that context, IL-15Rα binds and presents in *trans* IL-15 to a neighboring cell expressing the IL-2Rβ/γ_c_ complex [2]. At the 3D level, crystallographic data (PDB codes: 2Z3Q and 4GS7) of the IL-15/IL-15Rα complex exist, respectively with resolutions of 1.8 and 2.3 Å [10,11]. These data revealed that IL-15 residues located on B helix and AB and CD loops established key interactions with IL-15Rα. In the same vein, the resolution of the IL-15 heterotrimer (IL-15 with its three different receptor chains, corresponding to the full tetramer (quaternary complex): IL-15/IL-15Rα/IL-2Rβ/γ_c_) by X-ray diffraction [11] has shed light on the amino-acids participating in the interactions between the IL-15/IL-2Rβ and IL-15/γ_c_ interfaces. In agreement with those structures, direct mutagenesis studies have shown that, in the IL-15/IL-2Rβ interface, A and C helices play a major role, whereas the D helix is crucial for IL-15/γc binding. Such information has paved the way towards the development of specific immunotherapies based on IL-15 and IL-2 [12,13]. However, despite the obvious interest of these data, an accurate interpretation of the electron density in and around protein binding sites is often limited by the resolution. Furthermore, the inherent complexity of protein structures, and particularly of protein-protein assemblies, prevents a straightforward interpretation of the constitutive interactions at the three dimensional level [14,15,16]. Lastly, the X-ray crystallography provides a “static” structure, limiting the assessment of proteins’ functions and corresponding mechanisms of action.

In addition to structural and in vitro studies, molecular dynamics (MD) simulations are of prime interest to investigate at the atomic level the time-dependent behavior of the interleukin family members, in particular to get deeper insights within their structure and function relationships. In a recent study, MD simulations pointed out the key roles of the IL-6Rα chain in the assembly of the human IL-6 receptor complex [17], whereas a previous work, combining NMR measurements and MD calculations, had pointed out significant heterogeneity in terms of backbone fluctuations according to the IL-6 receptor epitopes [18]. Due to their structural similarity, IL-2 and IL-15 have often been simultaneously investigated combining (i) experiments to probe their structural (through X-ray crystallography) and/or affinity (with Surface Plasmon Resonance studies) features, and, (ii) molecular modeling, to reveal the major role of specific IL-2/IL-15 regions in the stabilization of particular receptor bound conformations [10,11]. Very recently, Baker and col. have engineered an IL-2 superkine using in vitro evolution studies in parallel with crystallographic, MD and affinity investigations on wild type IL-2 and selected mutants, highlighting, at the atomic level, the key amino-acid residues involved [19].

In the present work, we investigate different multimeric IL-15 models through MD simulations, in order to describe the structural and time related behavior of the various IL-15-receptor interfaces. To the best of our knowledge, our work is the first MD study of this important therapeutic target with a time length greater than 100 ns. In addition to the key features found by previous experimental works and further depicted here, new important components involved in the interface between IL-15 and its receptors are highlighted and described. This information could be useful for the design of new potent drugs targeting specifically these interfaces. Recent attempts have been carried out and the incorporation of dynamic features such as the ones highlighted in the present work could help to refine the models and improve the success of these strategies [20,21].

## 2. Methods

### 2.1. Structure Preparation

The initial coordinates for the molecular modeling study were extracted from the crystallographic data available on the Protein Data Bank for the IL-15/IL-15Rα binary complex (PDB ID: 2Z3Q) [10] and for the IL-15/IL-15Rα/IL-2Rβ/γc quaternary complex (PDB ID: 4GS7) [11]. Starting from these structures, four relevant combinations of IL-15 complexed with its receptors were built, in order to assess the influence of the different receptors on the overall IL-15 conformation. These systems were: the monomeric IL-15 form, the IL-15/IL-15Rα binary complex, the IL-15/IL-2Rβ/γc ternary complex and the full IL-15/IL-15Rα/IL-2Rβ/γc quaternary complex. Apart from the model consisting of IL-15 and its IL-15Rα, which was built starting from the 2Z3Q PDB file, all the other models were built starting from the 4GS7 PDB tetrameric structure. Indeed, since these two PDB entries remain the only ones available for IL-15, we think that their use as starting points for the relevant simulations is important, rather than using a single entry, in order to take into account their corresponding features, that might be related to the corresponding multimeric state. Nevertheless, we have computed the RMSD (considering Cα carbon atoms) of the IL-15/IL-15Rα chains in the dimer and tetramer crystallographic complexes. The value obtained, of 0.67 Å, clearly confirms that the two structures are similar. We are therefore confident that the protocol that we have used is safe.

The missing side-chain atoms, hydrogen atoms and disulfide bridges were added using the Prime tool in the Maestro program of the Schrödinger package [22]. A missing loop (residues 25–31 on IL-15) was added by homology modelling with an identical loop present on IL-2 (PDB ID 2B5I) using Prime from the Schrödinger Suite [22]. The glycosylated residues in the original PDB file were restored to their non-glycosylated form. All the water molecules located in the crystallographic structure at 4 Å of the interface residues were kept, as they might have an influence on the interactions established at the interfaces.

### 2.2. Molecular Dynamics Simulations

The MD simulations were carried out using the NAMD program [23]; all receptors complexes and counter-ions were described using the CHARMM36 force-field [24,25] and explicit TIP3P water molecules [26] were added to form a cuboid box of 12 Å dimensions around the protein. Each entire system was neutralized by the number of required counter-ions. These number were: 11, 6, 13 and 9 potassium ions for the monomer, dimer, trimer and tetramer species. Initially, two minimization steps were performed, each for 10,000 steps, wherein, at first, the water molecules and counter-ions were minimized while the protein was kept fixed and, finally, the whole system was minimized. The number of atoms and water molecules ranges from 30,736 (with 9652 water molecules) for the monomer to 190,645 (with 60,394 water molecules) for the tetramer. The cutoff distance, for all the steps, was set to 12 Å, with the switching function starting to take effect at 10 Å. The pair list distance, for which electrostatics and van der Waals interactions have been calculated during our calculations, were set to 14 Å. Being the size of the box used (distance of 12 Å between the solute and the wall of the box), we are confident that the that the protein did not interact with its images during the simulations, as proven by the values of the parameters of the MD analyses.

Following these steps, the various systems were heated to 300 K for 200 ps (NVT ensemble), at which point a 200 ns production step was performed within the NPT ensemble at 300 K. For the quaternary complex, three independent MD simulations of 100 ns each were run with identical starting structures but randomized velocities. All the aforementioned steps were performed with periodic boundary conditions, with the Langevin thermostat being used to keep the temperature constant and the Particle-Mesh Ewald (PME) method being used to treat non-bonded interactions with a cutoff of 12 Å [27]. The MD run was performed on a 2 fs timestep, with snapshots being saved every 2 ps (to a total of 100,000 snapshots for the full 200 ns). Energies were output every 0.25 ps. Bond lengths involving hydrogen atoms were fixed using the SHAKE algorithm and the non-bonded forces were calculated every step. The pressure was kept constant (1 bar) using a Langevin piston coupled to a heat bath, to keep the temperature constant at the aforementioned value.

### 2.3. MD Analyses

Root mean square deviation (RMSD) and Root mean square fluctuation (RMSF) values were obtained for IL-15 by first aligning the coordinates from all the corresponding systems to the IL-15 chain Cα atoms, using the first frame as reference, in the VMD plugin Trajectory Tool [28]. To complete this analysis, the matrices of all the possible RMSD value pairs during the whole 200 ns trajectory have also been calculated using the AmberTools18 collection of the Amber18 suite [29]. The RMSD values were calculated only accounting for Cα atoms of every residue, with the global value being further broken down into structural elements as follows: helix A (residues 1–19), A–B loop (residues 20–35), helix B (residues 36–54), helix C (residues 57–77), C-D loop (residues 78–95) and helix D (96–111). The B-C loop was not considered, as it is composed of only two residues and would not, as such, bring significant information to the analyses. It is worth noting that for these structural elements RMSD calculations, no re-alignment of the structures have been carried out, the values discussed being consequently free of roto-translations effects. The RMSF values presented are by-residue, with the RMSF value being used for each residue corresponding to the Cα RMSF value. In both cases, the values presented correspond to the last 100 ns of the MD simulations. As specified above for the structural elements RMSD calculations, no re-alignment has been carried out for the RMSF calculations.

The former analysis allows for a broad view of the conformational changes throughout the simulations, also serving as a tool for the validation of the models and verification of the equilibrated state of the different systems. The latter allows for a detailed view, on a per-residue basis, of the fluctuations observed throughout the simulations, thus allowing a deeper analysis of the influence of the flexibility of the residues on the system as a whole.

Following this preliminary analysis and confirmation of the equilibrated state of the system, the total number of contacts between IL-15 and each individual receptor was calculated, for each frame of the trajectory in the interval 100–200 ns, interval in which the trajectory was considered to be equilibrated for all cases. This was performed using the *nativecontacts* command of the *cpptraj* trajectory analysis tool, present in the AmberTools18 collection of the Amber18 suite [29]. A contact was thus defined based on a distance cut-off (<3 Å) between heavy atoms of each interface interacting amino acid residues. For this analysis, contacts present in both the reference frame, the 100 ns point of the simulation, and that were not present in this reference frame, were considered equally. The values reported in the supporting information for the various interfaces correspond therefore to average values considering the last 100 ns of the MD simulations. The choice of a value of 3 Å for this parameter is rationalized by the fact that we wanted to focus on the most specific interactions at the various interfaces. We have made several tries at greater distances, 3.5, 4 and 5 Å and decided to select 3 Å, with the weaker corresponding standard deviations.

Distance between residue pairs was analyzed based on the previously mentioned total number of contacts analysis. Individual (atom-atom pair) contacts were probed through the use of the aforementioned cpptraj tool, with all the atom pairs for each residue pair, at <3 Å from each other at any point during the simulation, being considered. The final distance taken into account was the minimum atom-atom distance in each frame at the 100–200 ns interval, for each residue pair. Further, average values of the atom-atom pair corresponding to the minimum distance were computed, as well as the percentage of the time wherein the residue-residue distance (as previously defined) remains at <3 Å. Lastly, the atoms considered when building the distance tables, as seen in Table S2, for instance, were the most prevalent throughout the simulation.

Water molecules residency time was calculated for the 100–200 ns interval, in each system, using the hbond command of the aforementioned cpptraj tool, with only inter-receptor (and hence, no intra-chain) solute-solvent hydrogen bonds being considered. The acceptor-donor heavy atom distance considered was 3.2 Å and all the bridging water molecules with more than 10% residency time (as defined by the ratio between the number of frames wherein each residue establishes a hydrogen bond with a non-specific water molecule versus the total number of frames) were kept. In other words, the residency time indicates the time in which any water molecule is bridged to receptor residues through inter chain hydrogen-bonds.

To complete these structural investigations, MM/GBSA calculations were realized, based on the MD trajectories obtained for each multimeric model. Let us consider the equation for the binding free energy of association:

$$\Delta G_{bind}=\langle G_{AB}\rangle -\langle G_{A}\rangle -\langle G_{B}\rangle$$

In the calculations, A and B were systematically different, depending on the multimeric system and the interface considered. As a matter of example, let’s consider the estimation of the binding free energy of association of the β interface for the trimeric and tetrameric complexes. For these calculations, B was the IL-2Rβ receptor chain, whereas A corresponded respectively to IL-15 in the trimeric complex and in the full tetramer. In other words, for these calculations, we have extracted, from the trimeric or full quaternary complex, the IL-15 monomer and the receptor chain under investigation in order to consider specifically in the calculations the corresponding binding energy between two chains. This method allowed to compare the free energy of binding of the different receptor chains with IL-15 but also to estimate the potential influence of the quaternary structure on this property. The averages used for the calculations correspond to the time interval between 100–200 ns, wherein the simulations were considered to be equilibrated.

## 3. Results and Discussion

### 3.1. The Quaternary Structure Impacts the Flexibility of the IL-15 Receptor Interfaces

First, we compare the RMSDs of various IL-15 components (whole chain, interfacial sites) as a function of the multimeric state of IL-15, in order to probe the effect of the quaternary structure on the interface dynamics. Table S1 and Figure S1 in the supporting information (SI) gathers the statistic parameters of the RMSD obtained and show the evolution of the IL-15 RMSD for the four models.

It appears from the results of this first global analysis that the flexibility of the IL-15 chain is strongly dependent on its quaternary structure. However, the trends have to be considered cautiously since for the majority of the systems considered, the average RMSD values are not significantly different. As such, we observed that the IL-15/IL-15Rα dimer is slightly more “restricted” (1.82 (0.20) Å) in terms of conformational flexibility, when compared to the free monomeric IL-15 (1.80 (0.36) Å), for which the range of RMSD values is larger. This result is in line with the previously mentioned analysis of the contacts established at the interface and the resulting loss of conformational freedom [10]. The most notable difference concerns the trimeric form (IL-15/IL-2Rβ/γc) which shows in particular lower structural stability (2.55 ± 0.48) Å) compared to the quaternary complex (1.91 (0.24) Å), highlighting the possible stabilizing effect of the binding of the IL-15Rα chain. However, it is worth noting that the IL-15 RMSD is in fact significantly weaker for the trimer when the Cα of the residues belonging to the C-D loop are removed (1.45 ± 0.15 Å compared to the initial 2.55 (0.48) Å), highlighting the particular flexibility of this loop and its influence (see Figure S2 in the SI).

**Figure 2:**
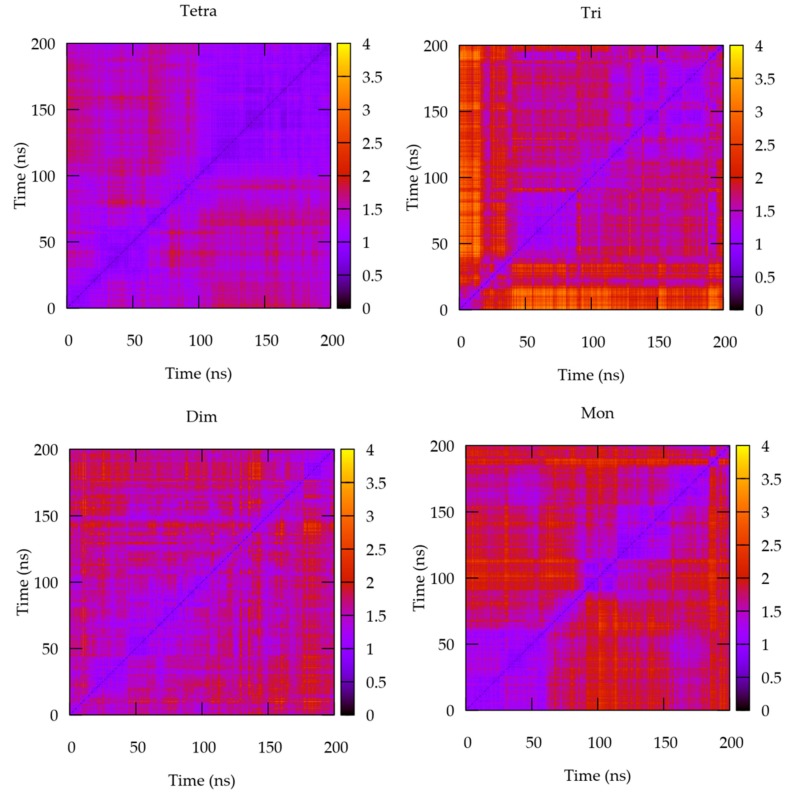
Matrices of all possible RMSD pairs computed from the trajectories of the four species considered (from the left to the right and from the top: tetramer; trimer; dimer and monomer) over the whole 200 ns of the simulations.

The matrices of all the possible RMSD value pairs during the trajectory have also been considered, since they have been reported to be more suited to evaluate the efficacy of the equilibration [30]. These data are shown in Figure 2 for the various complexed forms.

The visual inspection of these figures shows, in line with the previous analysis, that the four systems behave differently. Thus, it can clearly be seen that in the case of the IL-15/IL-15Rα/IL-2Rβ/γc tetramer, from about 100 ns, the complex adopts homogeneous conformations, conserved till the end of the simulation, in agreement with the equilibration of the system. In contrast, all the other models considered show regular changes of conformations along the simulation time. This behavior is expected for the monomer, which indeed shows the greatest conformational flexibility, being not constrained at any interface in comparison with the other models. The dimeric and trimeric complexes have an intermediate comportment, pointing out significant conformational changes along the simulation time.

It is worth noting that the duplicate and triplicate for the quaternary complex show very similar RMSD matrices profiles (Figure S3 in the SI) compared to the ones shown in Figure 2 (top left corner), reinforcing the trends pointed out for this system.

**Figure 3:**
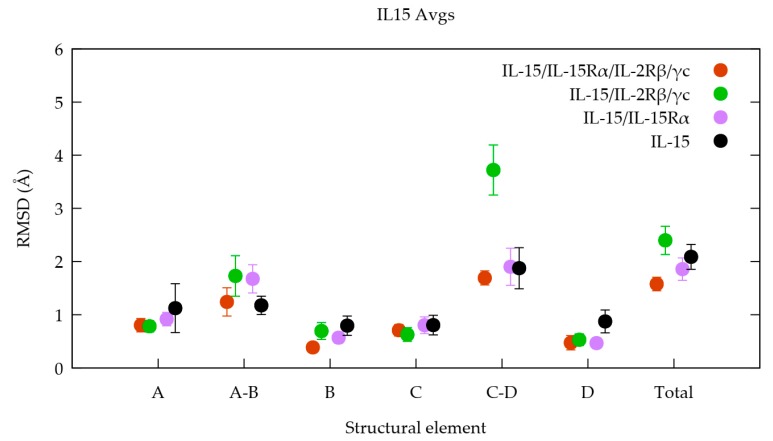
Average RMSDs values together with their standard deviations, calculated for each specific structural elements of the IL-15 chain over the last 100 ns of the MD simulations.

In order to gain deeper insights, we broke the RMSD analysis down into the specific IL-15 structural elements (see the methodology section). Figure 3 shows respectively their RMSD in the four heteromeric models for the last 100 ns of the simulation interval. The corresponding figure for the whole simulation (0–200 ns) is reported in the SI (Figure S4).

Through the analysis of Figure 3, different features are identified following the various structural elements considered (A-D helices and associated loops). It is clear that the largest standard deviation associated to the average RMSD value is, in all but one case (the only exception is loop C-D), obtained for the free IL-15 cytokine (black dots/lines in Figure 3), which is indeed expected to present the largest conformational freedom. Accordingly, we observed that for most structural elements, the smallest RMSD variation range is the one of the tetramer (orange in Figure 3), in agreement with the fact that this structure is the most constrained; all the IL-15 interfaces being involved in their corresponding contacts. The consideration of the evolution of the average RMSD values for the various systems, also pointed out interesting trends. However, since the differences are not statistically significant, these tendencies have to be considered cautiously. In fact, if we consider helix B, whose residues are in contact with the IL-15Rα receptor, RMSDs are decreasing in the expected order: IL-15 (0.80 ± 0.18 Å) > IL-15/IL-2Rβ/γc (0.69 ± 0.16 Å) > IL-15/IL-15Rα (0.57 ± 0.10 Å) > IL-15/IL-15Rα/IL-2Rβ/γc (0.39 ± 0.09 Å).

A similar behavior is apparent for helix A, which is known to participate in contacts with residues of the IL-2Rβ chain. Indeed, the corresponding order of evolution of the RMSD, despite not significant from a statistical point of view, is the following: IL-15 (1.12 ± 0.46 Å) > IL-15/IL-15Rα (0.93 ± 0.13 Å) > IL-15/IL-2Rβ/γc (0.78 ± 0.11 Å) ~ IL-15/IL-15Rα/IL-2Rβ/γc (0.80 ± 0.13 Å).

For this element, the forms with the largest RMSD value are the monomer and the IL-15/IL-15Rα dimer, for which helix A conserves its conformational flexibility, the interface being free. Similar trends could not be drawn from the consideration of the corresponding values for the A-B and C-D loops, which showed the largest amplitude of structural variations, as should be expected for loops. One behavior, however, deserves a special attention. Indeed, the C-D loop presents a large average RMSD (3.72 ± 0.47 Å) for the trimeric form compared to the other forms. In fact, this behavior can be rationalized by the fact that residues of this loop are involved in the IL-15Rα interface, remaining therefore exposed to the solvent in the IL-15/IL-2Rβ/γc trimeric receptor, thus compensating for the constraints imposed by the presence of the two other receptor chains. It is worth noting that if the whole simulation time length is considered, the RMSD average is even much larger (4.93 ± 1.15 Å, Figure S3) highlighting the particular flexible character of this loop.

#### RMSF

**Figure 4:**
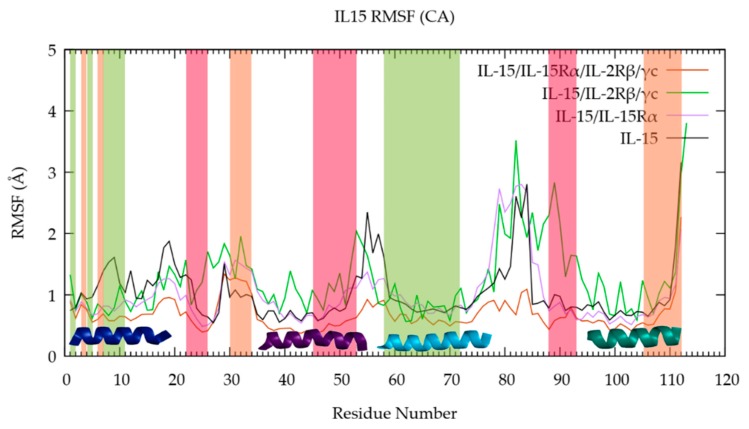
RMSF of Cα carbon atoms of IL-15 residues in the investigated multimeric models over the last 100 ns of the MD simulations. The shaded areas indicate the IL-15 interfaces residues interacting with IL-15Rα (red), IL-12Rβ (green), and γc (orange). Helices A (dark blue), B (purple), C (light blue) and D (teal) of IL-15 are also represented.

**Figure 5:**
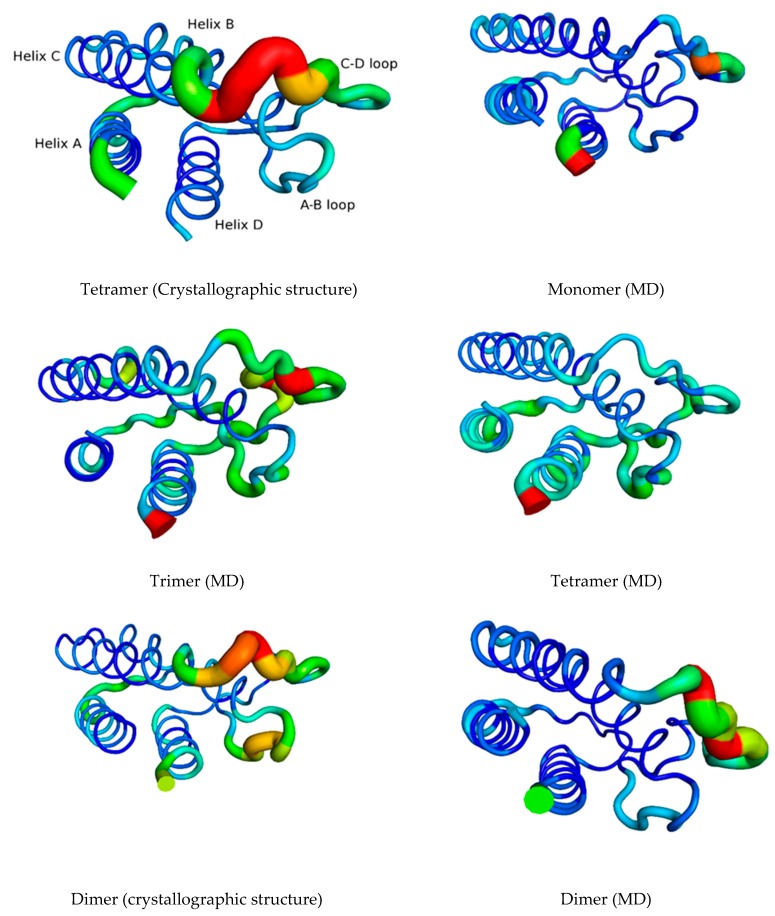
RMSF of Cα carbon atoms of IL-15 residues in the various investigated multimeric models considering the last 100 ns of the MD simulations. In the sake of comparison, the corresponding figures have been prepared for IL-15 in the two crystal structures, the thickness of the line being related to the B factor values. The scale for the RMSF is from 0 to 4 Å, corresponding to B-factor values from 0 to 440 Å^2^ (blue to red colors).

To get a complementary picture of the flexibility of the various IL-15 components pointed out in the previous section, we have then turned to RMSF analyses. Figure 4 shows the RMSF of Cα carbon atoms of IL-15 residues obtained for the various multimeric models considered and Figure 5 presents them graphically, in complement to the previous data, using the various IL-15 structures, for a better visualizing of its flexibility in the various forms considered. Figure S5 in the SI, which shows the corresponding RMSF profiles for the duplicate and triplicate, confirms the behavior of the full tetrameric complex.

Figure 4 and Figure 5 confirm the trends drawn from the RMSD analyses, since it is possible to denote a different flexible character according to the position of the amino acid residues, for the different studied models. Globally, the RMSF values appear larger for the free IL-15 structure and the trimeric complex (black and green lines), in coherence with the larger flexibility of the first as a monomer, being unrestricted at all interfaces, and the previous results. This behavior is seen on Figure 5 for both systems, the number of different zones and the thickness of the lines being more important for the monomer and the trimer. However, such a behavior is not observed for A-B and C-D loops.

The examination of the IL-15 specific structural elements in the three multimeric models also provides a complementary description to the previous RMSD analyses, evidenced by Figure 4 and Figure 5. As such, the position of the loops is clearly pointed out from the RMSF plot and highlighted on Figure 5, with a maximal RMSF value corresponding to the A-B (residues 20 to 36) and C-D (residues 78 to 96) loops of IL-15. For helix A, it appears that the largest RMSF values (corresponding to the thickest lines on Figure 5) are obtained for the free IL-15, compared to the trimeric complex IL-15/IL-2Rβ/γc (exhibiting the lowest value, i.e., the thinnest line on Figure 5) a trend which is consistent with the interaction of helix A residues with the IL-2Rβ chain [11]. Similarly, the dimeric complex (IL-15/IL-15Rα) exhibits higher RMSF values for helix A, but they are lower than for the free IL-15, suggesting a slight stabilizing effect of IL-15Rα. The situation is different for helix B, since the largest RMSF values are found for the IL-15/IL-2Rβ/γc trimer, followed by similar values for the free IL-15 and the IL-15/IL-15Rα dimer, with the lowest values being obtained for the tetrameric model. This behavior is in agreement with the fact that helix B residues are known to be involved in the interface with the IL-15Rα chain [10]. It is worth noting that a specific region corresponding to the end of helix B (from 52 to 54) and the B-C loop (residues 55 and 56) shows large values in the trimer and the monomer, corresponding to thicker lines on Figure 5. For helix C, a similar profile than the one obtained for helix A could be discerned, presenting an increased conformational stability. For helix D, only the free IL-15 behaves differently from the three other species. Indeed, on average, the RMSF values are significantly higher for the IL-15 monomer compared to the three other models, which behave very similarly. Lastly, it is remarkable that for both A-B and C-D loops, the largest structural fluctuations are obtained for the trimeric IL-15/IL-2Rβ/γc receptor, in agreement with the fact that those residues are involved in contacts with IL-15Rα chain amino acids and therefore, with full conformational freedom in the trimer.

### 3.2. The Quaternary Structure Impacts the Structural and Energetic Features of the Various Interfaces

We then compare the number of contacts and the energetic features at the various interfaces between IL-15 and its receptors as a function of the investigated multimeric models. Table 1 clearly evidences different number of contacts and binding free energies for each interface. Indeed, the average number of contacts involving the IL-15Rα chain is higher than 40, whereas the corresponding values are around 30 and 20 for the IL-2Rβ and γc chains, respectively. It is worth noting that for the quaternary complex, the calculation of the number of contacts for two replica led to similar values, strengthening the trends pointed out. Thus, for the duplicate, the various contacts amount to 38, 32 and 26 for the IL-15Rα, IL-2Rβ and γc chains whereas for the triplicate, 37, 30 and 27 contacts are predicted. From a binding free energy perspective, the MM/GBSA results support these trends, since the value computed for the IL-15/IL-15Rα interface, of about −80 kcal/mol is significantly greater than the corresponding data for the IL-2Rβ (−28 kcal/mol) and γc (−16 kcal/mol) chains, respectively.

Such differences can be correlated to the higher affinity of IL-15 for the IL-15Rα chain (between 7−40 pM) compared to the corresponding quantity for IL-2Rβ/γc complexes (30 nM) [31,32,33]. In the rest of the manuscript our reference system will be the tetramer.

### 3.3. Highlighting Novel Key Structural Features at IL-15 Interfaces

In order to get deeper details from our MD simulations, the different interface features were then scrutinized using a pairwise amino acid analysis of the interactions across the various interfaces. These results are reported in the supporting information (Tables S2–S4). For the sake of comparison, we have determined using the same methodology the number of contacts at the different interfaces in the crystal structures. To clearly distinguish the information originating from the crystal structure analyses and from our MD simulations a color code has been used (experimental: yellow; MD: blue) in the Tables of the supporting information. It can be seen from this first comparison that for the IL-15/IL-15Rα interface, only two contacts, on a total of 34, are brought to light by our MD simulations. In the case of the IL-15/IL-2Rβ interface, the corresponding data are 7 and 29. Lastly, for the IL-15/γc interface, 12 interactions predicted by the MD simulations, on a total of 19 contacts, were not reported in the crystal structure. The same analyses were carried out on replicates and support these structural features, with the majority of interactions, both in terms of types of contacts and with respect to the residues involved, being conserved between the different simulations for the quaternary complex.

**Figure 6:**
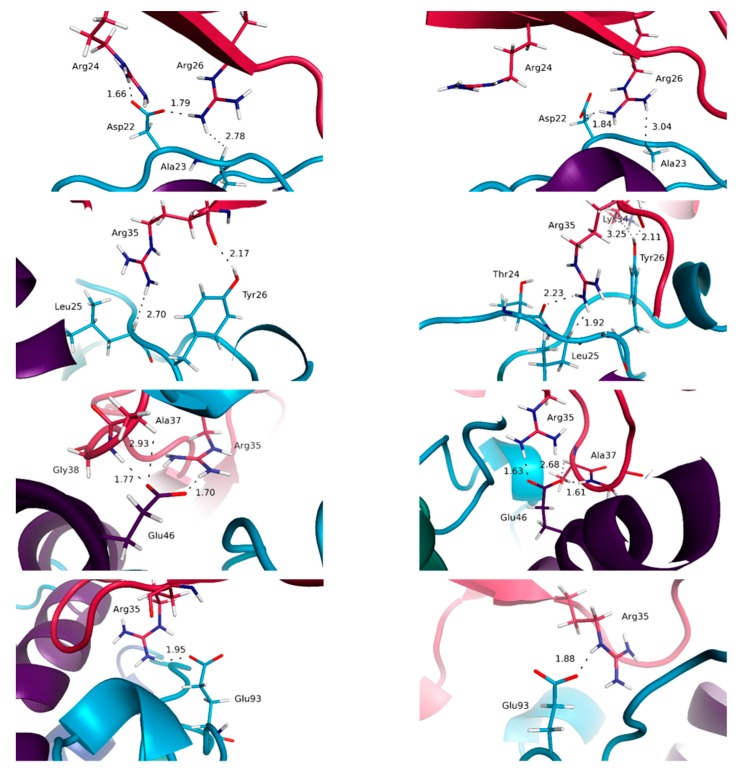
Representation of key interactions at the IL-15/IL-15Rα interface for the full system (**left**) and the dimeric system (**right**). The distances, indicated in Å, are represented by dotted lines.

Interestingly, the majority of the interactions having the highest percentages of occurrence within our simulations are obtained for the IL-15/IL-15Rα interface. Indeed, the average percentages of occurrence of the various interactions for this interface are of 78 and 86%, respectively, for the dimeric and tetrameric complexes, whereas the corresponding values are 64 and 67%, and 45 and 39%, for IL-15 interfaces with IL-2Rβ and γc chains in trimeric and tetrameric complexes. In agreement with the crystallographic structures, both in the dimeric (2Z3Q [10]) and tetrameric (4GS7 [11]) forms, the key role played by charged residues of IL-15 is evidenced by our simulations. Particularly, the salt bridges between Asp22 (A-B loop), Glu46 and Glu53 (helix B) of IL-15 with the positively charged lateral chains of Arg26 and Arg35 on the IL-15Rα receptor were virtually conserved throughout the whole simulation (percentages higher than 97%) (Figure 6).

Moreover, short hydrogen-bonds occurring during the full time length of the calculations are worth noticing. They correspond to interactions between the phenolic OH group of Tyr26 (A–B loop of IL-15) with the main carbonyl chain of Arg35 (IL-15Rα) and the carboxylate group of Glu53 (helix B of IL–15) with the alcohol OH group of Ser40. We also note the occurrence of Van der Waals interactions between apolar amino acid side chains present on each side of this interface that proved to be conserved all along the simulation time (interactions between methylene groups of the lateral chains of Glu53 (helix B) and Glu89 (C–D loop) of IL-15 with Leu42 and Ile64 of IL-15Rα, respectively). The agreement between our theoretical results and the experimental data for the IL-15/IL-15Rα interface, more precisely Asp22, Glu46 and Glu53 of IL-15 [32], Glu46 of IL-15 [34], and Arg35 of IL-15Rα [35], makes us confident in the interest of our model. The first contact not reported in the earlier crystallographic analyses concern a salt bridge (carboxylate of Asp22 on IL-15 with one ammonium NH of Arg24 on IL-15Rα) present at 87% along the simulation length in the tetramer. The second one involves the aliphatic lateral chain of Leu52 (IL-15), in close contact with its counterpart on the IL-15Rα side, corresponding to the lateral chain of Leu42, this contact being predicted in the trimeric complex with a percentage of about 73%. For the IL-15/IL-15Rα interface, our simulations bring limited new information. Nevertheless, our data confirm the relative importance of amino acid residues at the interface and clarify their role at the atomic level, in particular through their high percentage of occurrence along the simulation time. It is interesting to note that all the interactions discussed above are systematically observed both in the dimer and the full receptor, with very similar features.

**Figure 7:**
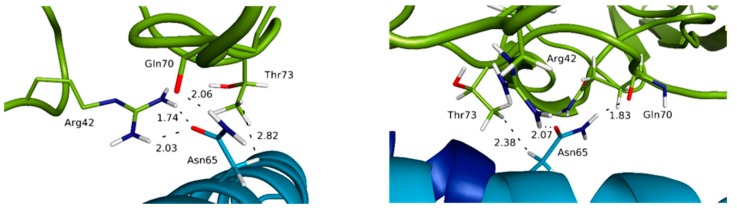
Representation of key interactions at the IL-15/IL-2Rβ interface for the full system (**left**) and the trimeric system (**right**). The distances, indicated in Å, are represented by dotted lines.

For the IL-15/IL-2Rβ interface, noticeable differences are obtained compared to the IL-15/IL-15Rα interface. First, as already mentioned, the percentage of occurrence of the various contacts is significantly lower compared to the IL-15/IL-15Rα interface, in line with the moderate affinity of this complex [36]. As a matter of fact, while the number of contacts in the trimer and tetramer forms remains similar, the interactions tend to be less conserved, the chemical fragments involved in the trimeric complex being in some situations different to the ones in interaction in the tetramer. Remarkably, among the various interactions observed, salt bridges are much less numerous for this interface than in the case of the IL-15/IL-15Rα. Indeed, only one interaction of this kind is observed, between the carboxylate group of Asp61 (helix C of IL-15) and the ammonium lateral chain of Lys71 (IL-2Rβ). The most frequent interactions are hydrogen bonds. Two residues of IL-15 appear to play a pivotal role in such inter-chain hydrogen-bond interactions: Asp8 (helix A) and Asn65 (helix C). Indeed, one hydrogen-bond is kept all along the simulation time, and involves the carboxylate group of IL-15 Asp8 and the phenolic OH of IL-2Rβ Tyr134 (Figure 7).

Another one, with a significant occurrence (about 45% of the simulation time) and not reported previously, concerns the OH of IL-15 Ser7 (helix A) and the carboxylate group of IL-2Rβ Glu136. Interestingly, the amide group of the Asn65 lateral chain uses both its hydrogen-bond donor and acceptor potential through the NH_2_ group (with the main carbonyl of Gln70), and the C=O (with the ammonium of Arg42) fragments. Furthermore, one of the methylene groups of the lateral chain of Asn65 is in van der Waals contact most of the simulation (99.7% and 98.6% in the trimer and the full tetramer, respectively) with a methylene group of Thr73, this interaction being never mentioned before. The importance of these residues has already been highlighted by mutagenesis studies: for IL-2, Asp20 [37], Asn88 [38] and IL-15, Asp8 [39], Asn65 [34]. In addition, Ile68 and Leu69 residues of IL-15 are predicted to be in van der Waals contacts (through fragments of their aliphatic chain) with a significant occurrence (from 35 to 98%) along the simulation time with several IL-2Rβ residues (Lys41, Arg42, Thr73, Thr74, Val75). Among those, some (Ile68 (IL-15) with Lys41 (IL-12Rβ); Ile68 (IL-15) with Thr73 (IL-12Rβ)) have not been previously discussed. In these cases, the fragments involved in the various contacts are not always identical in the trimer and the tetramer, highlighting the higher flexibility of these groups, in line with the weaker specificity of such interactions.

**Figure 8:**
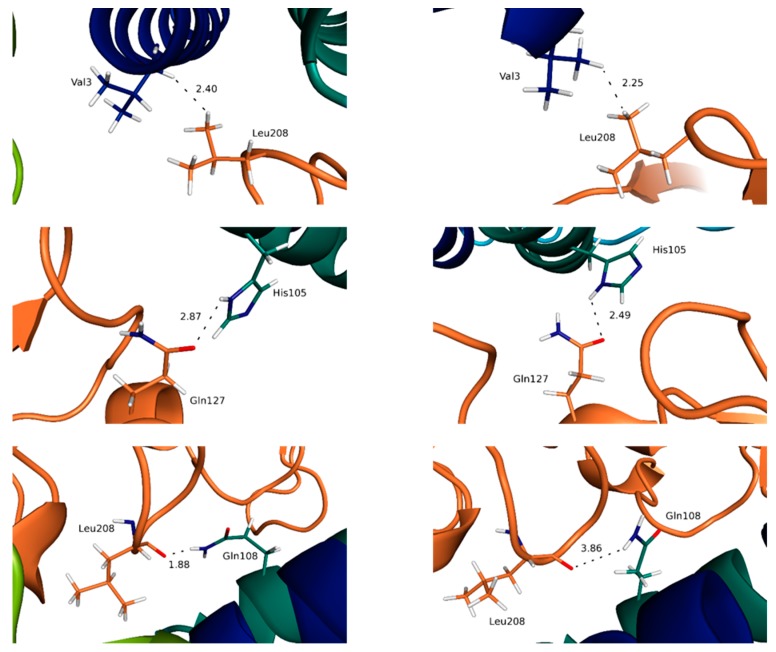
Representation of key interactions at the IL-15/γc interface for the full system (**left**) and the trimeric system (**right**). The distances, indicated in Å, are represented by dotted lines.

The IL-15/γc complex appears clearly to be the least stabilized, with significantly lower occurrence of the interactions highlighted, compared to the other IL-15 interfaces, in line with the difficulty to measure the affinity of this complex [36]. As a consequence, the differences obtained for the trimer and the full tetramer are the most significant, as well as the difference between the static (crystallographic) results and the ones from the MD simulations. Two residues of IL-15 are involved in hydrogen bond interactions at this interface: His105 and Gln108, both belonging to helix D (Figure 8).

It is worth mentioning that the contribution of His105 at this interface has not been evidenced in the crystallographic analysis. His105 interacts through the NH of the imidazole ring as hydrogen bond donor, the carbonyl oxygen of Gln127 (γc) behaving as the hydrogen bond acceptor, Gln108 through the NH_2_ group of the amide lateral chain with the main chain carbonyl oxygen of Pro207 (γc). The respective occurrence of these interactions, of 51 (His105:Gln127) and 48% (Gln108:Pro207) highlight, nevertheless, substantial structural rearrangements, in line with the flexibility of this interface. The importance of residue Gln108 for both IL-15 and IL-2 (Gln126) has been confirmed by mutagenesis studies [39,40], and described in the earlier crystallographic structural analyses [11,41]. In line with the flexibility of this interface mentioned above, the MD simulations bring to light the contribution of the lateral chain of Ile111 (IL-15) which appears in weak interaction with the carbonyl oxygen of the Leu208 backbone (for 78%) in the tetrameric complex whereas it is interaction with the corresponding aliphatic lateral chain in the trimer (for 35%). In the same vein, the lateral chain of Asn112 (IL-15) is shown to switch between several different interactions (ranging from 10 to 25%) with residues of the γc chain (Tyr103, Asn158 and Leu208) during the MD simulations.

### 3.4. Water Molecules Stabilize the Interfaces

From previous crystallographic studies, the importance of water molecules in the high affinity values between IL-15 and some of its receptor chains, especially IL-15Rα, has been emphasized [10]. In this work, we have therefore explored the interactions of water molecules with surrounding residues at the vicinity of the various interfaces (see the methodology section). Table S5 (supporting information) presents the results obtained for the various interfaces in the various multimeric species.

**Figure 9:**
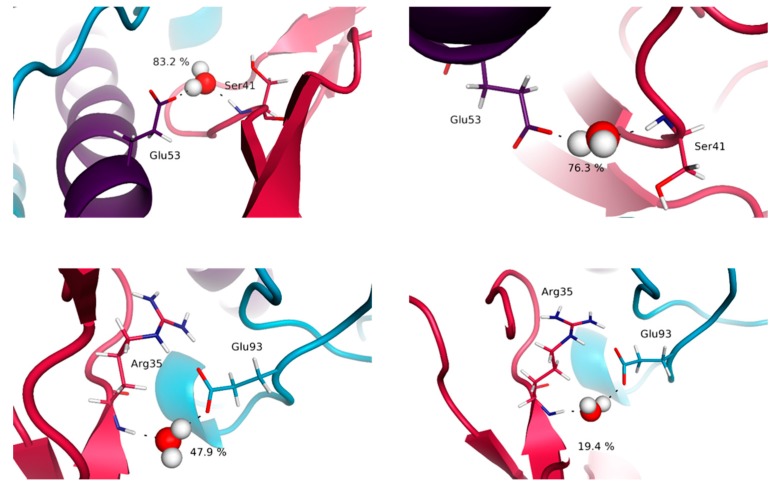
Representation of key bridging water molecules at the IL-15/IL-15Rα interface for the full system (**left**) and the dimeric system (**right**). The values indicated correspond to the percentage of presence of the water molecules along the simulation time.

The trends highlighted by our results are in concordance with the ones pointed out through the other descriptors. Indeed, it appears that the highest percentages of presence of water molecules are obtained for the IL-15/IL-15Rα interface, in line with the very high affinity reported for this complex [31,32] and the previously suggested key role of two water molecules. Interestingly, our results show a significant difference according to the quaternary structure since in the dimer, the number of contacts is significantly higher (12 instead of 8); some of the contacts being more frequent in the full system (around 83% of the simulation time) than in the dimer. Among the various hydrogen-bond networks established around these “conserved” bridging water molecules, a special role should be assigned to the one involving Glu53 (helix B) of IL-15 and Ser41 of IL-15Rα (Figure 9). Indeed, this specific water molecule coincides with the position occupied by a water molecule with a particularly low B factor in the crystallographic structure of the IL-15/IL-15Rα [10], this position being conserved, with a higher percentage (83 instead of 76%), in the full receptor. It is worth noting that the second position appearing for this interface as the most “occupied” by a water molecule corresponds to another important region of the IL-15/IL-15Rα interface, involving charged residues (Glu93:Arg35 (almost 50% in the full receptor) and Glu89:Lys36, around 20%) and reported in the experimental studies [10,11].

**Figure 10:**
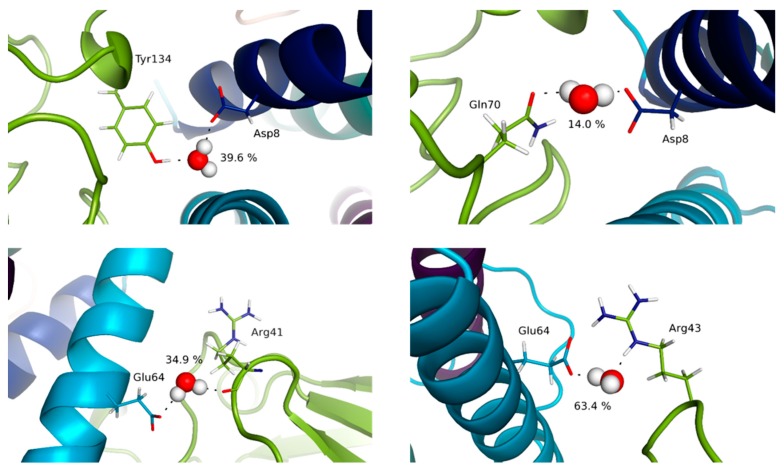
Representation of key bridging water molecules at the IL-15/IL-2Rβ interface for the full system (**left**) and the trimeric system (**right**). The values indicated correspond to the percentage of presence of the water molecules along the simulation time.

Remarkable features can also be pointed out for the IL-15/IL-2Rβ interface. In fact, bridging water molecules have previously been evidenced in the IL-2/IL-2Rβ interface through His133 and Tyr134 of IL-2Rβ interacting with Asp20 of IL-2 from X-ray crystallography studies [41]. Despite the resolution of the quaternary IL-15/IL-15Rα/IL-2Rβ/γc complex, no discussion on the specific behavior of water molecules at the various interfaces was carried out by Garcia and coworkers [11]. Therefore, our MD simulations provide a first way to probe their role. For the IL-15/IL-2Rβ interface, among the contacts with the highest occupancy, His133 and Tyr134 were actually involved (Asp8:Tyr134 (40%); Lys11:His133 (38%)), bridging water molecules involving these residues being predicted by the MD simulations, both in the trimer and the tetramer. However, it was surprising to see that the number of contacts at the IL-15/IL-2Rβ interface appears to be significantly higher (14 compared to 8) in the quaternary IL-15 complex than in the IL-15/IL-15Rα interface. Although the number of contacts is higher for this interface, the same residues are involved, which evidences a higher dynamic character of the interface, which could be in line with a lower affinity of the IL-15/IL-2Rβ complex compared to IL-15/IL-15Rα (Table S5). Another region, involving other polar and/or charged residues of both chains (IL-15: Asp61, Thr62 of helix C; IL-2Rβ: Arg42, Ser69, Gln70), and not mentioned by relevant experimental studies, is revealed by our MD simulations. This area involves other polar and/or charged residues of both chains (IL-15: Glu64; IL-2Rβ: Arg41, Arg43). Figure 10 shows as an example the one with the highest percentage, between Glu64 of IL-15 and Arg43 of IL-2Rβ, reaching 64% in the trimer, and Arg41 of IL-2Rβ, amounting to about 35% in the tetramer.

**Figure 11:**
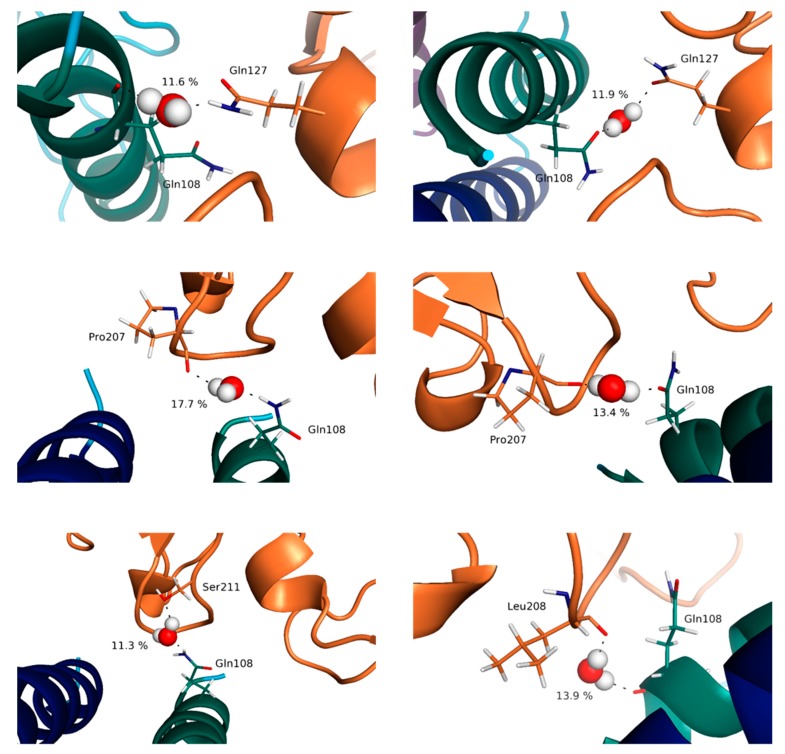
Representation of key bridging water molecules at the IL-15/γc interface for the full system (**left**) and the trimeric system (**right**). The values indicated correspond to the percentage of presence of the water molecules along the simulation time.

Lastly, both in terms of number of contacts and of occupancy percentage, the water molecules at the IL-15/γc appear much less conserved, in agreement with the weaker stability of this interface. However, there are relevant positions, which should be highlighted. Precisely, Gln108 interacts with a plethora of different residues located on the γc receptor, with relevant interactions with Pro207 (15 and 13% for the two respective systems) and Gln127 (12% for both systems) being evidenced (Figure 11). Furthermore, there is a water molecule mediating the interaction of the aforementioned residue (Gln108) with Leu208 exclusively in the trimeric system (14%) and with Ser211 exclusively in the tetrameric system (11%), further suggesting an important role of this residue in the establishment of the IL-15/γc interface. These results corroborate the ones outlined in the previous section.

## 4. Conclusions

In this study, new structural features of IL-15 interfaces (IL-15/IL-15Rα, IL-15/IL-2Rβ and IL-15/γc) in relevant IL-15 multimeric states have been reported on the basis of MD simulations. It is important to note that despite the fact that our study investigates the structural features of the various relevant (from a biological point of view) multimeric states of IL-15, namely the full quaternary complex: IL-15/IL-15Rα/IL-2Rβ/γc, the trimeric complex IL-15/IL-2Rβ/γc, the dimeric IL-15/IL-15Rα and monomeric IL-15 forms, the starting geometries used come from the experimental structures available, that is the full quaternary complexes (4GS7 pdb entry for the quaternary, trimeric complexes and the monomer) and the binary IL-15/IL-15Rα complex (2Z3Q pdb entry for the dimer). While this methodology might have limitations, we have not found any other alternative in the absence of the relevant corresponding experimental information. It is also worth noticing that with the 200 ns simulation time length used in this work, some potentially important dynamic fluctuations might be missed. Despite these limitations, nevertheless, significant new insights on the structural and dynamic behavior of this important therapeutic target have been highlighted in this work.

The RMSD analysis has allowed us to point out the significant influence of the quaternary structure on the variation in the receptor chains backbone. As such, the lowest structural stability (larger RMSD) was obtained for the trimeric form (IL-15/IL-2Rβ/γc), putting into evidence significant conformational changes. However, the global analysis suggests that the IL-15/IL-15Rα dimer seems to be more “constrained” in terms of conformational flexibility compared to the free monomeric IL-15, in line with the contacts established at the interface and the resulting loss of conformational freedom. The RMSD analysis of specific IL-15 structural elements brought light on more details. For all structural elements (A, B, C and D helices and loops), the smallest RMSD variation range was predicted for the tetramer, appearing as the most stable, due to the interactions of interfacial residues in all the possible interfaces, the largest RMSD being predicted for the monomeric IL-15. The consideration of individual RMSD values of the various structural elements has revealed interesting complementary trends. Notably, helix A, which possesses residues in interaction with residues of the IL-2Rβ chain, presents the largest RMSD value (1.99 ± 0.33 Å) in the IL-15/IL-15Rα dimer, for which these amino acids conserve their conformational flexibility. Similarly, for helix B, known to have residues in contact at the interface with the IL-15Rα receptor, the highest RMSD (1.78 ± 0.46 Å) was observed in the trimeric structure (IL-15/IL-2Rβ/γc). However, no significant trends have been suggested through a comparable analysis for C and D helices. In the same vein, the A-B and C-D loops showed the largest amounts of flexibility, which was not significantly different between the different systems, preventing the observation of a particular behavior.

The RMSF values for the various systems have been shown to be significantly larger for the free IL-15 chain as a whole, in line with the largest conformational flexibility of this state. The position of the loops has been unambiguously identified by the RMSF analysis, the maxima of the plots corresponding to the A-B (residues 20 to 36) and C-D (residues 78 to 96) amino acids. The behavior of the amino acid residues of the A-D helices complement the RMSD analyses, highlighting the influence of the multimeric state on the structural fluctuations at the various interfaces.

The consideration of the number of contacts established at the various interfaces have allowed us to highlight the notable larger number of contacts at the IL-15/IL-15Rα interface, followed by the IL-15/IL-2Rβ interface, the one with the lower number of interactions being the IL-15/γc interface.

The consideration of the pairwise amino acid interactions across the three interfaces for the three multimeric systems have allowed us to validate our model, with the key residues previously underlined by crystallographic analyses appearing as the ones with the highest percentages of presence along the simulation timecourse. Furthermore, our study provides a detailed chemical and structural atomistic description, through the measurement of the corresponding distances. Interestingly, the implication of residues, or fragments of residues, not reported previously through crystallographic analyses of IL-15 complexes, is delineated by our investigation. Specifically, for the IL-15/IL-15Rα interface, new hydrogen-bonds involving the IL-15 Glu46 and Glu89 known residues are highlighted by our work. Moreover, a salt bridge between the IL-15 Glu93 and the IL-15Rα Arg35 has been pointed out. For the IL-15/IL-2Rβ interface, the specific behavior of Asn65 was underlined, since the whole potential of interaction, specific and unspecific of the lateral chain of this amino acid appeared to be exploited in interactions at this interface. As such, the amide chain uses both its hydrogen-bond donor and acceptor potential through the NH_2_ group (with the main chain carbonyl of the Gln70 of IL-2Rβ), and the C=O (with the ammonium of the IL-2Rβ Arg42) fragments, one methylene group of the aliphatic chain being in close contact (around 98% of the simulation time) with a methylene group of the IL-2Rβ Thr73. Lastly, for the IL-15/γc interface, despite the fact that the corresponding complex seems to be the least stabilized, the role of important residues was suggested, notably through the implication of their lateral chain in contacts across the interface. On the IL-15 side, His105 and Gln108, presented contacts for upwards of 70% of the simulation time.

Lastly, the important role of water molecules was evidenced. The previous crystallographic investigations have emphasized this importance for the IL-15/IL-15Rα interface, whereas we were able to complement and build upon these data by showing that the same behavior was suggested for the IL-15/IL-2Rβ and IL-15/γc interfaces, with water molecules being involved in specific networks pertaining some of the key residues at the interfaces.

Together, our results allowed us to expand on conclusions previously established through experimental investigations, mainly through X-ray crystallography, and provide a detailed description, at the atomic level, of pivotal features of IL-15 interfaces. This knowledge should be useful for the design of specific modulators of these important PPIs.

## Figures and Tables

**Table 1:** Number of contacts and MM/GBSA binding free energies (kcal/mol) for the various interfaces in the three multimeric (dimer, trimer and tetramer) forms of IL-15. The number in parentheses corresponds to the standard deviation.

		Number of Contacts			Δ*G*_bind_		
Interface	Model	Dimer	Trimer	Tetramer	Dimer	Trimer	Tetramer
IL-15/IL-15Rα		43 (4)	-	41 (3)	−80.3 (6.6)		−83.8 (8.2)
IL-15/IL-2Rβ			28 (4)	26 (4)		−29.1 (5.6)	−27.2 (6.6)
IL-15/γc			18 (5)	18 (7)		−16.2 (7.7)	−17.3 (10.5)

## Supplementary Materials

The following are available online at https://www.mdpi.com/1420-3049/24/18/3261/s1, Table S1: RMSD Statistics (min, max, average and corresponding standard deviation) for the various IL-15 multimeric models; Figure S1: RMSDs plots of the Cα carbon atoms of the whole IL-15 chain over 200 ns of MD simulations. Figure S2: RMSDs plots of the Cα carbon atoms of the IL-15 chain with (brown) and without (green) the Cα atoms of the C-D loop over 200 ns of MD simulations. Figure S3: Average RMSDs values together with their standard deviations, calculated for each specific structural elements of the IL-15 chain over 200 ns of MD simulations. Table S2: Residues, atoms and corresponding distances and percentage of presence along the simulation time for the interface contacts predicted by the MD simulations for the IL-15/IL-15Rα complex in the dimeric and tetrameric receptors; Table S3: Residues, atoms and corresponding distances and percentage of presence along the simulation time for the interface contacts predicted by the MD simulations for the IL-15/IL-2Rβ complex in the trimeric and tetrameric receptors; Table S4: Residues, atoms and corresponding distances and percentage of presence along the simulation time for the interface contacts predicted by the MD simulations for the IL-15/γc complex in the trimeric and tetrameric receptors; Table S5: Percentage, along the simulation time, of water molecules in hydrogen-bond interactions with amino acid residues across the various interfaces of IL-15.
