# Supplementary material for: Mechanistic and Structural Insights on the IL-15 System through Molecular Dynamics Simulations
Source: Molecules. 2019 Sep 6;24(18):3261. doi: 10.3390/molecules24183261 (PMC6767322; doi:10.3390/molecules24183261)
Supplement: Supplementary file 1 [file molecules-24-03261-s001.pdf]

Supplymentary Materials

Table S1. RMSD Statistics (min, max, average and corresponding standard deviation) for the various IL-15 multimeric models.

Model Statistics	IL-15	IL-15/IL-15Rα	IL-15/IL-2Rβ/γc	IL-15/IL-15Rα/IL-2Rβ/γc
Min	0.77	0.72	0.63	0.90
Max	2.70	2.50	3.40	2.43
Average	1.85	1.82	2.55	1.91
Std[1]	0.36	0.20	0.48	0.24

[1] Standard deviation

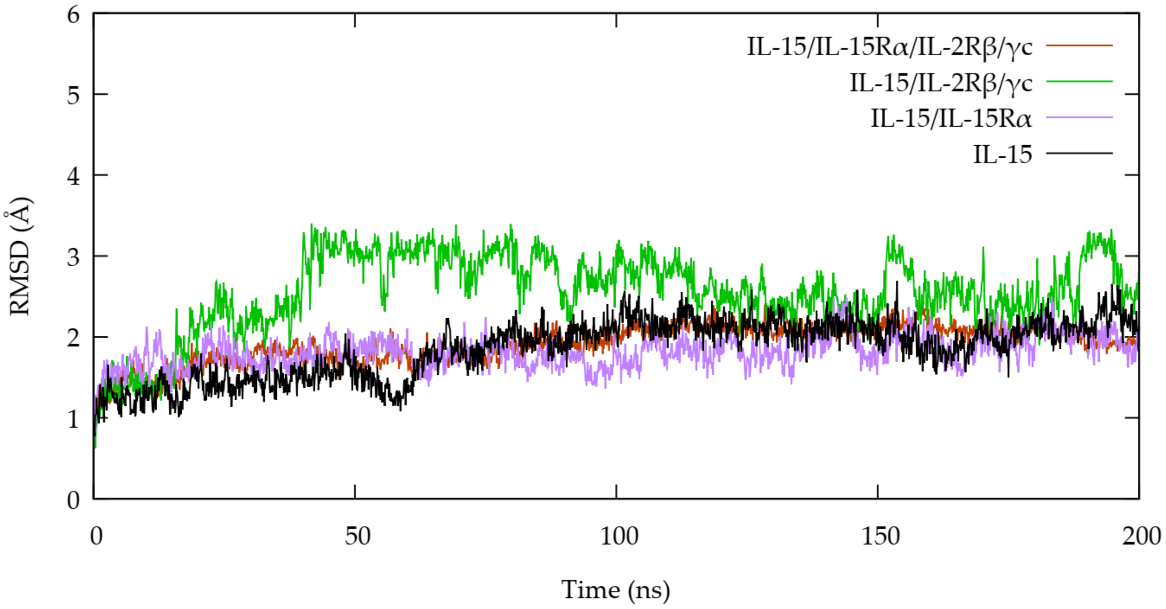

Figure S1. RMSDs plots of the Cα carbon atoms of the whole IL-15 chain over 200 ns of MD simulations.

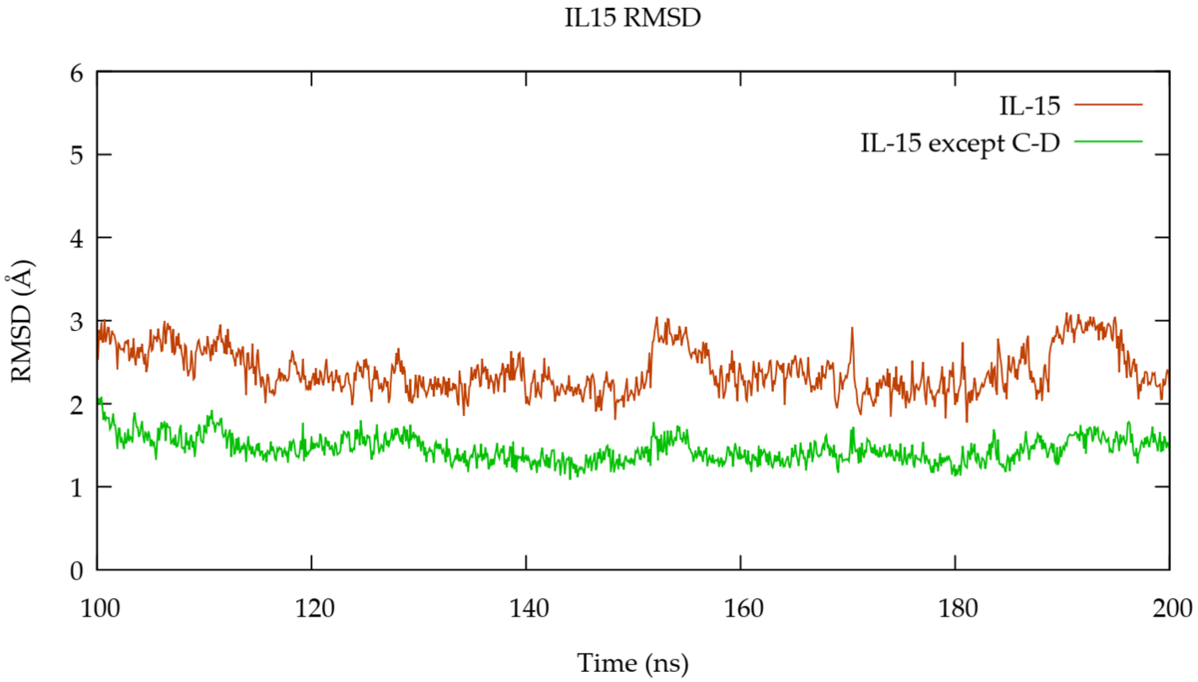

Figure S2. RMSDs plots of the Cα carbon atoms of the IL-15 chain with (brown) and without (green) the Cα atoms of the C-D loop over 200 ns of MD simulations.

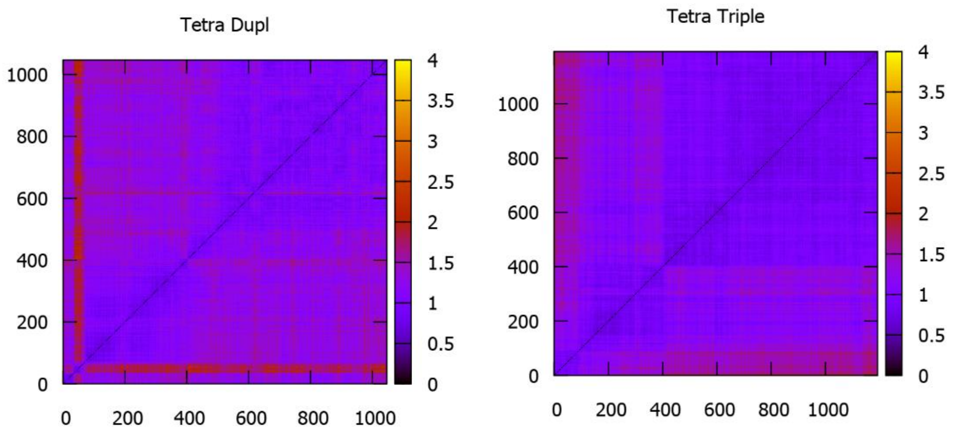

Figure S3. Matrices of all possible RMSD pairs computed from the trajectories of the duplicate and triplicate of the tetramer (100 ns of MD simulations). The ordinate and abscissa axes correspond to the number of frames.

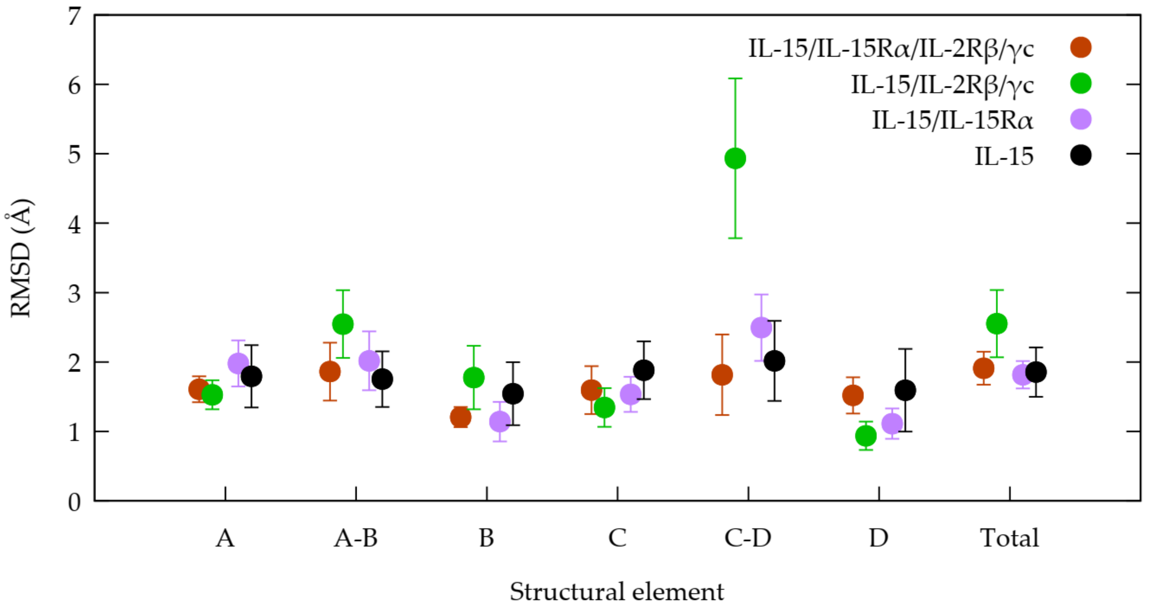

Figure S4. Average RMSDs values together with their standard deviations, calculated for each specific structural elements of the IL-15 chain over 200 ns of MD simulations.

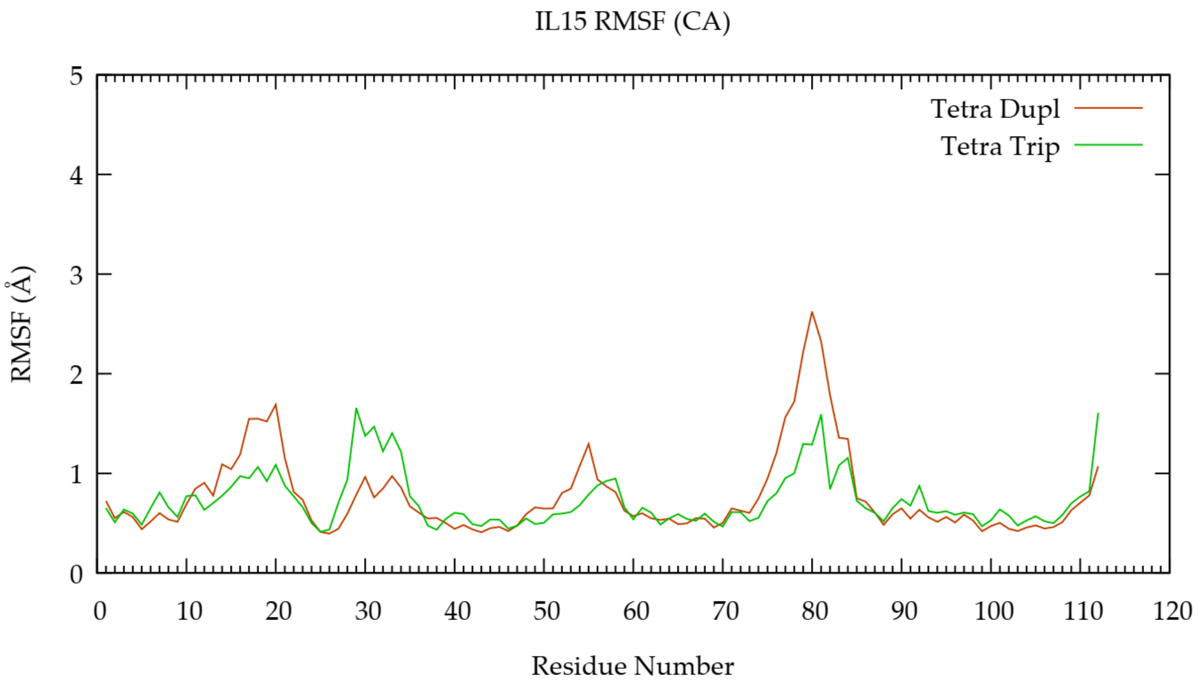

Figure S5. RMSF of Cα carbon atoms of IL-15 residues for duplicate and triplicate of the MD simulations of the tetramer considering the first 100 ns.

Table S2. Residues, atoms and corresponding distances and percentage of presence along the simulation time for the interface contacts predicted by the MD simulations for the IL-15/IL-15Rα complex in the dimeric and tetrameric receptors. The lines colored in yellow and blue correspond respectively to the contacts observed in the crystallographic structures and revealed by the MD simulations. The distances reported are average distances for the last 100 ns of the MD simulation, meaning that for a given interatomic distance, the value has been inferior to 3.0 Å during a certain time of the simulation. When a given average was superior to the limit of 3.0, it has been reported in italic in the table.

				**Dimer**	**Tetramer**	**Dimer**	**Tetramer**
**IL-15**		**IL-15Rα**		**d(H…A) Å**	**d(H…A) Å**	**Percentage (%)**	**Percentage (%)**
**Residue**	**Atom**	**Residue**	**Atom**				
Asp22	OD2	Arg24	HH11		2.27(1.42)		87
Asp22	OD1	Arg26	(NH1)H	1.83 (58)	1.74(16)	97	100
	CB(HB)	Arg26	(NH2)H	2.83(42)	2.80(33)	73	73
Thr24	(C)O	Arg35	(NH2)H	2.68(47)	2.98(41)	74	50
Leu25	(CA)HA	Arg35	(NH2)H	2.36(20)	2.45(20)	100	100
Tyr26	O(H)	Lys34	CB(HB)	2.71(24)	2.97(32)	89	59
Tyr26	(O)H	Arg35	O(C)	1.81(13)	1.79(12)	100	100
Tyr26	(CD2)H	Ala37	(CB)HB2		2.49(23)		98,00
Tyr26	(CD2)H	Ala37	(CA)HA	2.52(22)		98	
Leu45	HD22	Ala37	CB(HB1)		2.48(37)		94
Leu45	(CB)HB	Ala37	CB(HB3)	2.61(41)		84	
Leu45	(C)O	Gly38	(CA)HA2	2.54(21)	2.44(20)	98	99
Glu46	OE1	Arg35	(NH2)H	1.66(07)	1.66(07)	100	100
Glu46	OE2	Ala37	(CA)HA	2.55(25)	2.49(22)	96	98
Glu46	OE2	Gly38	HN	1.81(12)	1.78(12)	100	100
Gln48	(CB)HB2	Gly38	(CA)HA2	2.81(47)	3.66(94)	72	26
Val49	(CG2)HG22	Arg35	(NH1)HH12	2.70(34)	2.69(30)	84	84
Val49	(CG2)HG22	Gly38	(CA)HA2	2.35(25)	2.42(26)	98	97
Val49	(CG2)HG22	Thr39	(C)O	2.93(29)	2.89(27)	64	70
Val49	(CG2)HG11	Ser40	(CB)HB2	2.39(26)	2.22(22)	98	99
Leu52	(CB)HB2	Ser40	(CB)HB2	2.76(48)	2.59(34)	73	88
Leu52	(CD2)HD22	Leu42	(CB)HB2	2.93(75)		73	
Leu52	(CD2)HD22	Ser60	(OG)HG1		2.78(47)		76
Glu53	(C)O	Arg24	(NH1)HH12	3.35(1.35)		40	
Glu53	OE1	Arg26	(NH2)H22	1.67(08)	1.67(08)	100	100
Glu53	OE2	Ser40	(OG)HG1	1.95(32)	1.88(19)	99	100
Glu53	(CG)HG2	Leu42	(CB)HB1	2.41(27)	2.42(23)	98	98
Cys88	(CB)HB1	Ala37	(CB)HB2	2.41(28)	2.67(29)	98	87
Glu89	(CB)HB1	Lys34	(CE)HE1	3.34(1.28)	2.25(35)	51	98
Glu89	(CG)HG2	Arg35	(C)O	3.06(39)	3.10(47)	51	49
Glu89	(CG)HG2	Lys36	(CA)HA	1.93(36)	1.91(34)	99	99
Glu89	OE2	Ala37	HN	1.94(21)	2.00(19)	100	100
Glu89	(CG)HG2	Ile64	(CD)HD3	2.41(30)	2.37(28)	96	96
Glu90	(CA)HA	Lys34	HZ2	4.10(1.90)	2.50(84)	33	70

Glu90	(CG)HG2	Pro67	(OG)HG1	4.37(2.25)		49	
Glu93	OE1	Arg35	(NE)HE	1.83(25)	2.07(75)	98	87

Table S3. Residues, atoms and corresponding distances and percentage of presence along the simulation time for the interface contacts predicted by the MD simulations for the IL-15/IL-2Rβ complex in the trimeric and tetrameric receptors. The lines colored in yellow and blue correspond respectively to the contacts observed in the crystallographic structures and revealed by the MD simulations. The distances reported are average distances for the last 100 ns of the MD simulation, meaning that for a given interatomic distance, the value has been inferior to 3.0 Å during a certain time of the simulation. When a given average was superior to the limit of 3.0, it has been reported in italic in the table.

				**Trimer**	**Tetramer**	**Trimer**	**Tetramer**
**IL-15**		**IL-2Rβ**		**d(H…A) Å**	**d(H…A) Å**	**Percentage (%)**	**Percentage (%)**
**Residue**	**Atom**	**Residue**	**Atom**				
Asn4	(ND2)HD22	Thr74	(OG1)HG1	3.21(1.20)		59	
Asn4	(ND2)HD21	Tyr134	(CB)HB1	2.68(23)	2.73(61)	93	83
Ser7	(OG)HG1	His133	(CB)HB2	2.87(51)		68	
Ser7	(CB)HB1	Tyr134	(CD2)HD2	2.95(67)	*6.21(3.20)*	66	*32*
Ser7	(OG)HG1	Glu136	OE2		*3.70(2.15)*		*45*
Asp8	OD1	Tyr134	(OH)HH	1.85(39)	2.57(97)	96	71
Lys11	(CB)HB2	His133	(CD2)HD2	3.15(1.15)		55	
Asp61	OD2	Leu69	(CG)HG1	2.18(82)	*3.15(1.58)*	82	*54*
Asp61	(CB)HB1	Gln70	(C)O	*3.27(45)*	*3.14(47)*	*83*	*47*
Asp61	OD2	Lys71	(NZ)HZ1	1.82(33)	1.92(75)	98	93
Glu64	OE1	Arg42	(NH1)HH11	2.18(60)	2.36(88)	85	80
Asn65	OD1	Arg42	(NH1)HH12	1.87(16)	1.85(13)	100	100
Asn65	(ND2)HD22	Gln70	(C)O	2.05(24)	2.16(32)	100	98
Asn65	(CB)HB1	Thr73	(CH)HG23	2.34(21)	2.44(21)	100	99
Asn65	(CB)HB1	Tyr134	(OH)HH	*3.52(51)*	*3.57(1.26)*	*15*	*45*
Ile68	(CD)HD2	Lys41	(CG)HG1		2.64(47)		85
Ile68	(CG2)HG21	Lys41	(CD)HD2	2.37(25)		98	
Ile68	(CD)HD2	Arg42	NH2		*3.25(58)*		*35*
Ile68	(CD)HD3	Arg42	(CD)HD1	*3.12(61)*		*50*	
Ile68	(CG2)HG21	Thr73	OG1		*3.19(40)*		*35*
Ile68	(CG2)HG23	Thr73	(OH1)HG1	*3.11(45)*		*48*	
Ile68	(CG2)HG21	Val75	(CB)HB		2.44(25)		97
Ile68	(CG2)HG22	Val75	(CG2)HG22	2.81(37)		74	
Leu69	(CD2)HD21	Thr73	(CB)HB		2.87(89)		73
Leu69	(CB)HB2	Thr73	(CG2)HG23	2.58(33)		88	
Leu69	(CD)HD12	Thr74	(OG1)HG1	*3.53(1.63)*		*51*	
Leu69	(CA)HA	Val75	(CG2)HG22	2.46(36)	2.44(32)	90	94
Leu69	(CD1)HD12	Tyr134	(OH)HH		*4.30(2.28)*		*34*
Asp72	(CB)HB2	Val75	(CG1)HG12		2.35(21)		99

Table S4. Residues, atoms and corresponding distances and percentage of presence along the simulation time for the interface contacts predicted by the MD simulations for the IL-15/γc complex in the trimeric and tetrameric receptors. The lines colored in yellow and blue correspond respectively to the contacts observed in the crystallographic structures and revealed by the MD simulations. The distances reported are average distances for the last 100 ns of the MD simulation, meaning that for a given interatomic distance, the value has been inferior to 3.0 Å during a certain time of the simulation. When a given average was superior to the limit of 3.0, it has been reported in italic in the table.

				**Trimer**	**Tetramer**	**Trimer**	**Tetramer**
**IL-15**		**γc**		**d(H…A) Å**	**d(H…A) Å**	**Percentage (%)**	**Percentage (%)**
**Residue**	**Atom**	**Residue**	**Atom**				
Val3	(CA)HA	Leu208	(CD2)HD22		2.83(62)		67
Val3	(CG2)HG22	Leu208	(CD2)HD22	*3.05(91)*		*63*	
Ile6	(CG2)HG21	Pro207	(CB)HB2		*4.13(1.46)*		*33*
Ile6	(CD1)HD1	Leu208	(CA)HA		*3.95(1.53)*		*39*
Val31	O	Asn71	(ND2)HD21		*5.64(2.07)*		*11%*
Val31	O	Asn71	(CB1)HB1	*4.01(1.67)*		*32%*	
His32	(CA)HA	Asn71	(CB1)HB1	*3.12(1.57)*		*71%*	
Pro33	(CD2)HD2	Gln104	(NE2)HE21	*3.78(1.40)*		*42%*	
His105	(CE1)HE1	Thr105	O	*3.20(1.64)*		*69%*	
His105	NE2	Lys125	(CE)HE1		*3.63(1.08)*		*37%*
His105	NE2	Lys125	(NZ)HZ1	*3.95(1.99)*		*45%*	
His105	(ND1)HD1	Gln127	OE1	*3.63(1.64)*		*51%*	
His105	NE2	Asn128	(ND2)HD21		*4.02(2.30)*		*52%*
Gln108	(CB)HB1	Tyr103	(CE1)HE1	*3.08(87)*	*7.56(2.81)*	*55%*	*13%*
Gln108	(CB)HB1	Gln127	(NE2)HE22		*3.33(1.17)*		*49%*
Gln108	OE1	Gln127	(NE2)HE22	2.42(61)		85%	
Gln108	(NE2)HE22	Pro207	O	*4.50(1.47)*	*3.61(1.68)*	*11%*	*48%*
Gln108	(CG)HG2	Leu208	O	*3.02(95)*	*3.00(1.21)*	*63%*	*76%*
Gln108	(CB)HB1	Cys209	(CA)HA		*3.60(1.22)*		*36%*

Table S5. Percentage, along the simulation time, of water molecules in hydrogen-bond interactions with amino acid residues across the various interfaces of IL-15.

**Bridged amino acid residues**			
**IL-15**	**IL-15Rα**	**% in the dimer**	**% in the tetramer**
Glu53	Ser41	76	83
Glu93	Arg35		48
Glu53	Glu44		42
Asp24	Arg26		28
Glu53	Arg24	52	26
Asp22	Arg24	34	
Asp22	Arg26	31	
Glu89	Ala37	22	19
Glu89	Lys34	22	15
Glu93	Arg35	19	
Glu89	Lys36	18	20
Glu92	Lys34	16	
Glu89+Leu91	Lys34	14	
Glu90	Pro67	13	
Tyr26+Glu93	Arg35	11	
**IL-15**	**IL-2β**	**% in the trimer**	**% in the tetramer**
Glu64	Arg43	63	
Asn4	Tyr134	47	19
Asp8	Tyr134		40
Asn1	Thr74	38	17
Ile68	Arg41	36	
Glu64	Trp44	26	
Lys11	His133	26	38
Glu64	Arg41		35
Lys11	Asp68	23	
Asp61 + Glu64	Arg42		16
Asn4	Gln188		13
Asp8	His133		13
Ser7	His133	15	13
Asn1+Asn4	Thr74	15	
Asn72	Arg41	14	
Asp8	Gln70	14	
Glu64	Arg42	13	
Asp61	Arg42	12	
Asp61	Ser69	12	12
Asp61	Lys71	11	13
Glu64	Lys71	11	
Asp61	Ala66	11	
Thr62	Gln70	10	12
Asp61	Gln70		11
Glu64	Arg42		11
**IL-15**	**γc**	**% in the trimer**	**% in the tetramer**
Gln108	Leu208	14	
Gln108	Pro207	13	18
His105	Gln104	12	
Gln108	Gln127	12	12

His105	Tyr103	11	
His105+Gln108	Gln127	10	
Gln108	Ser211		11
Val31	Asn71		11
His107	Gln127		11
